# Cardiac Septal Metastasis From Poorly Differentiated Colorectal Adenocarcinoma: A Rare Case Illustrating the Role of Multimodal Imaging and Multidisciplinary Management

**DOI:** 10.1155/cric/6626317

**Published:** 2025-11-30

**Authors:** Ameer Odeh, Danish Saleh, Robert Bayer, Douglas R. Johnston, Iman Imanirad, Mohamed Al-Kazaz

**Affiliations:** ^1^Feinberg School of Medicine, Northwestern University, Chicago, Illinois, USA; ^2^Division of Cardiology, Bluhm Cardiovascular Institute, Chicago, Illinois, USA; ^3^Division of Hematology & Oncology, Northwestern Memorial Hospital, Chicago, Illinois, USA; ^4^Division of Cardiac Surgery, Northwestern Memorial Hospital, Chicago, Illinois, USA; ^5^Moffitt Cancer Center Magnolia Campus, Tampa, Florida, USA

## Abstract

**Background:**

Cardiac metastases from colorectal adenocarcinoma are rare and may present with varied or absent symptoms.

**Case Summary:**

A middle-aged woman with recurrent colorectal adenocarcinoma presented with an infiltrating ventricular septal cardiac mass. Multimodal imaging, including PET-CT and cardiac MRI, was crucial for both identifying the lesion as a metastasis and for subsequent serial monitoring. Extensive cardiac involvement necessitated specialized surgical and radiation oncology expertise at a high-volume center, though extra-cardiac lesions led to deferring surgery or radiation as therapeutic options. Ultimately, lesion regression was achieved with intensive chemotherapy and immunotherapy targeting the primary malignancy.

**Discussion:**

To our knowledge, no prior cases have documented colorectal adenocarcinoma metastasizing to the interventricular septum with interatrial extension. Management of intracardiac metastases is complex and requires a multidisciplinary approach.

**Take Home Messages:**

Accurate diagnosis and characterization of cardiac masses requires a multimodal imaging approach, while effective management depends on a multidisciplinary strategy tailored to treatment goals and patient-specific factors. Surgical resection can be considered at high-volume centers but may be deferred in metastatic disease if it does not improve prognosis or symptoms. Factoring in the expected response to medical therapy is also important.

## 1. History of Presentation

A 56-year-old woman was previously diagnosed with poorly differentiated colorectal adenocarcinoma with mucinous and signet ring features. She underwent a right hemicolectomy in 2017, followed by eight cycles of chemotherapy with capecitabine and oxaliplatin (CAPOX) and bevacizumab, completed in 2018. That same year, resection of a mediastinal mass confirmed metastatic colorectal adenocarcinoma. In 2020, she experienced peritoneal recurrence and was treated with six cycles of leucovorin, fluorouracil, and oxaliplatin (FOLFOX), followed by cytoreductive surgery and hyperthermic intraperitoneal chemotherapy (CRS-HIPEC) with Mitomycin-C in 2021. The cytoreductive procedure included ileocolostomy anastomosis resection, bilateral salpingo-oophorectomy, peritonectomy, omentectomy, and excision of peritoneal nodules followed by three cycles of leucovorin, fluorouracil, and irinotecan (FOLFIRI) and cetuximab.

**Figure 1:**
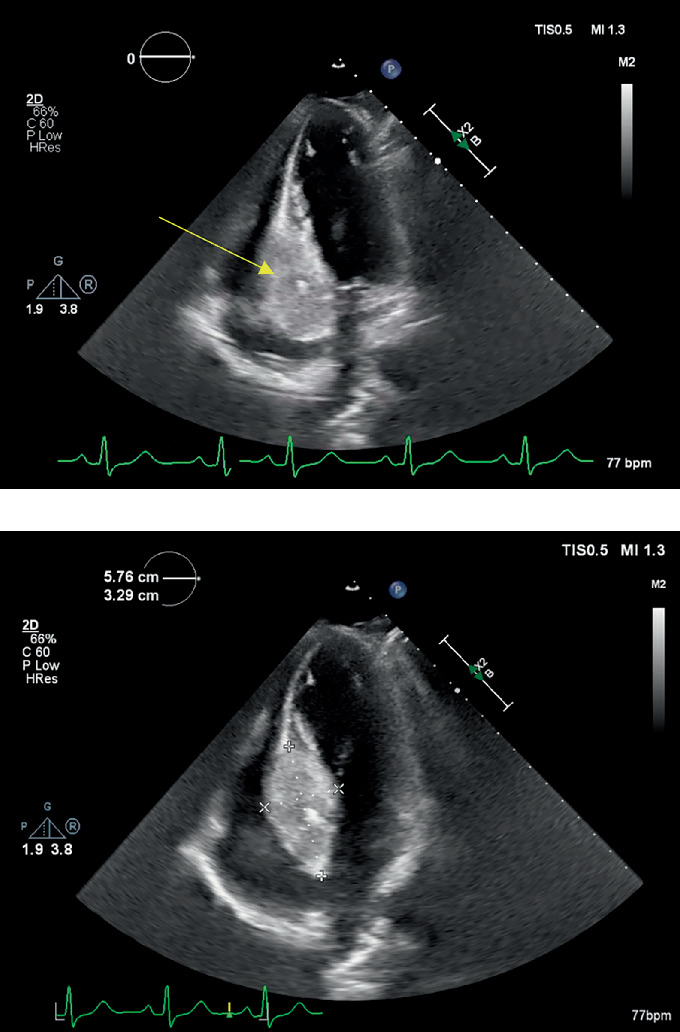
Transthoracic echocardiogram performed at an outside institution 2 years prior to presentation. (a) Apical 4-chamber view with arrow pointing to intracardiac mass located at the ventriculo-atrial septal junction, (b) measuring roughly 5.8 cm × 3.3 cm.

**Figure 2:**
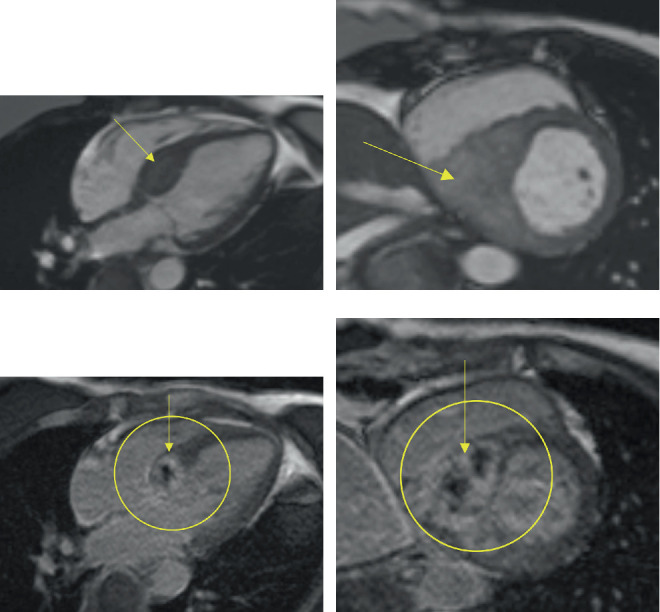
Cardiac MRI performed at an outside institution 2 years prior to presentation. (a) Four-chamber view and (b) short-axis view demonstrating a large mass within the interventricular septum. (c) Four-chamber view and (d) short-axis view postgadolinium enhancement showing central hypointensity within the mass, likely due to necrosis.

She presented in July 2021 with shortness of breath and chest pain at an outside hospital. Exam revealed normal vital signs, regular rate and rhythm, no murmurs at rest or with Valsalva, no lower extremity edema, and no jugular venous distension. Transthoracic echocardiogram revealed an echogenic structure at the interventricular/interatrial septal junction (5.8 cm × 3.3 cm; Figure 1 and cardiac MRI (cMRI) confirmed a 5.6 × 5.3 cm infiltrative mass involving the left ventricular septum and anterior wall, with extension into the superior interatrial septum (Figure 2). Endomyocardial biopsy was nondiagnostic, showing focal subendocardial scarring, prominent endothelial cells, and scattered interstitial edema. Immunohistochemical staining did not provide definitive evidence of carcinoma. However, blood work showed elevated circulating tumor DNA (ctDNA) levels, indicating the presence of residual colorectal adenocarcinoma.

She presented to our center 2 years later for a second opinion on the management of her cardiac mass.

## 2. Past Medical History

Cardiac history is significant for sick sinus syndrome requiring pacemaker placement in July 2021, and paroxysmal atrial fibrillation. Patient's history was also notable for malignancy-associated pulmonary embolism, multiple sclerosis, and anxiety/depression.

### 2.1. Differential Diagnosis

The differential diagnoses for the cardiac mass included metastatic disease from colorectal cancer, primary cardiac neoplasm, hypertrophic cardiomyopathy, endocarditis, and infiltrative diseases (e.g., amyloidosis).

### 2.2. Investigations

**Figure 3:**
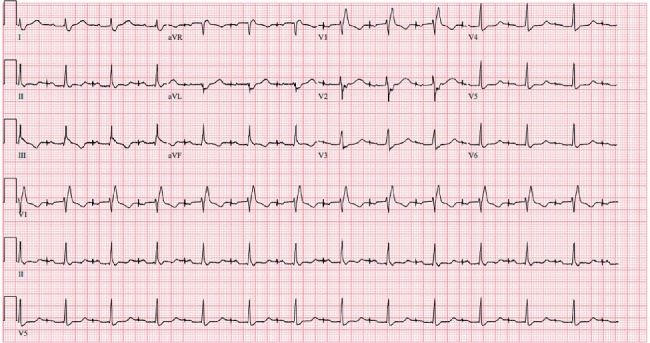
Baseline electrocardiogram. Notable for an atrial-paced rhythm, first-degree AV block, and a right bundle branch block.

**Figure 4:**
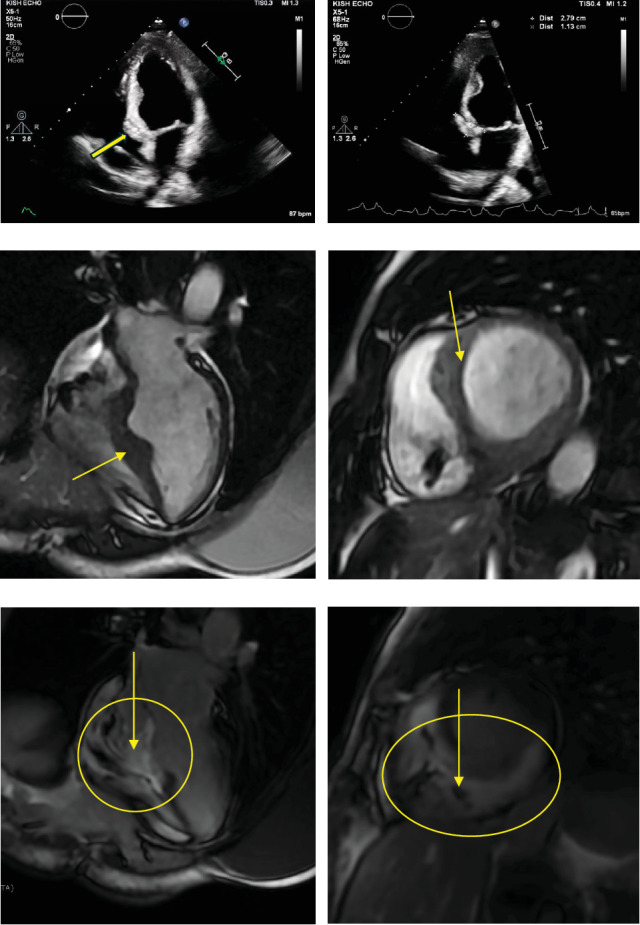
Cardiac mass characterization on transthoracic echocardiography and cardiac MRI. (a, b) Transthoracic echocardiogram performed on initial presentation to our institution: (a) Apical 4-chamber view showing an intracardiac mass at the ventriculo-atrial septal junction. (b) Measuring roughly 2.8 × 1.1 cm. (c–f) Cardiac magnetic resonance imaging for further mass characterization: (c) Four-chamber view demonstrating asymmetric thickening in the interventricular septum and aneurysmal akinetic region at the basal inferoseptum (yellow arrow). (d) Short-axis view with asymmetric septal thickening within the interventricular septum (yellow arrow). (e) Postgadolinium four-chamber view revealing dense late gadolinium enhancement (LGE) involving basal to mid septum and inferior wall, with extension into the anterior wall. (f) Postgadolinium short-axis view showing dense LGE and central hypointensity in the inferoseptum, consistent with necrosis (yellow arrow).

Baseline EKG obtained during the initial visit showed an atrial-paced rhythm, first-degree AV block, and a right bundle branch block (Figure 3). Her transthoracic echocardiogram revealed an echogenic structure at the interventricular and interatrial septal junction consistent with her history of an intracardiac lesion/mass (Figure 4). Otherwise, left ventricular ejection fraction was 45%, with no signs of left ventricular outflow tract (LVOT) obstruction at rest or with Valsalva.

**Figure 5:**
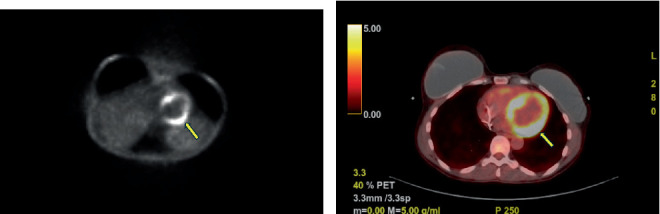
F-18 fluorodeoxyglucose (FDG) PET CT scan. (a) Two-dimensional nonattenuation correction (NAC) image and (b) color scale image demonstrate diffuse uptake of FDG in the left ventricular myocardium with increased asymmetric uptake in the posterior left ventricle and left ventricular septum as noted by the yellow arrow.

Contrast-enhanced CT of the chest, abdomen, and pelvis demonstrated a 6.1 × 4.5 cm mass involving the inferior portion of the interventricular region and base of the left ventricle, with relatively low attenuation. In addition, the CT scan revealed a new ~2 cm periumbilical soft tissue mass, with IR-guided biopsy confirming metastatic colorectal adenocarcinoma. cMRI ordered for further lesion characterization identified a 2.3-cm akinetic region of myocardium at the inferior right ventricular insertion, involving the basal inferoseptum and basal inferior wall. Postgadolinium imaging showed high signal intensity on late gadolinium enhancement across the basal to mid left ventricle, extending to the right ventricular septum and inferior right ventricular wall. Central hypointensity and necrosis were present, consistent with an intracardiac mass (Figure 4). The left ventricular ejection fraction was estimated at approximately 45%. PET-CT revealed a hypermetabolic, hypodense mass along the posterior aspect of the left ventricular septum, consistent with cardiac metastasis (Figure 5).

### 2.3. Management

The patient received multiple chemotherapy and immunotherapy regimens for her colorectal cancer and peritoneal metastases, with reasonable response over the years. At the time of evaluation at our institution, the patient was receiving ipilimumab and nivolumab, which she continued, though further progression occurred. Periumbilical biopsy confirmed recurrent peritoneal mucinous adenocarcinoma with signet ring cell features, compatible with her primary colorectal carcinoma. In the setting of progression of disease, the patient was transitioned to panitumab monotherapy.

In assessing the intracardiac mass, the first priority was to evaluate its hemodynamic impact, including the potential for LVOT obstruction and the risk of necrosis leading to intracardiac shunting. Transthoracic echocardiography was a valuable tool for this assessment and was reassuring in both respects. Given the patient's clinical history and elevated ctDNA levels without other radiographic evidence of disease, the mass was felt to be metastatic from her primary colorectal adenocarcinoma rather than a primary cardiac tumor. A multidisciplinary team was then engaged to determine the best approach, weighing surgical versus medical management. Although complex cardiac resection with reconstruction might have been technically feasible at our high-volume center, its overall benefit was uncertain in the context of a recent periumbilical biopsy confirming recurrent peritoneal mucinous adenocarcinoma. As a result, surgical intervention was deferred, and medical therapy was adjusted accordingly.

Radiation therapy was also deferred due to the high risk of radiation-induced cardiotoxicity, lack of significant cardiac symptoms/dysfunction, and the presence of extra-cardiac metastases. The intracardiac mass was monitored with serial imaging while the primary colorectal malignancy was being treated to ensure stability and offer supportive cardiac care. Further cMRIs and CT scans showed that the intracardiac mass remained stable with panitumab infusions. Regular appointments with the cardiology and oncology teams showed stable cardiovascular status.

### 2.4. Outcome and Follow-Up

As of April 2025, the patient remains on palliative panitumumab monotherapy with stable disease. A CT scan performed in January 2025 demonstrated no change in the size of the cardiac tumor. Transthoracic echocardiography in April 2025 also showed a stable intracardiac mass without evidence of obstruction on Valsalva. No additional atrial or ventricular arrhythmias were detected on continued rhythm monitoring. Given the absence of cardiac symptoms, the current plan is to continue serial imaging to monitor the mass and provide supportive cardiac care as needed, while continuing to manage her primary colorectal malignancy.

## 3. Discussion

Primary cardiac tumors are rare, with cardiac metastasis occurring approximately 30 times more frequently than a primary cardiac neoplasm [1]. One Italian study of 18,751 autopsies found heart metastases in 9.1% of cancer cases [2]. Among 1066 colon carcinoma cases, 13 (1.2%) had cardiac metastases. Colorectal metastases to the heart are not well documented in the literature, likely due to the rarity of the condition. Common primary malignancies that metastasize to the heart include lung, breast, esophageal cancers, and lymphomas [3]. Metastases may involve any cardiac layer and chamber, with some reports suggesting a predilection for the ventricles [2]. Clinical presentation varies widely, from incidental findings to constitutional symptoms, heart failure, arrhythmias, systemic embolization, tamponade, or sudden death [1].

To our knowledge, no cases of interventricular septal metastasis from colorectal cancer have been previously described. Prior reports have documented right-sided [4–8] and left atrial [9, 10] metastases. Our patient had an unusual site of metastasis, with extension from the LV septum to the interatrial septum. Despite the mass size, she had mild symptoms, with improvement in shortness of breath after pacemaker placement suggesting sinus node dysfunction. The chest pain was likely multifactorial secondary to the presence of systemic disease, history of pulmonary emboli, and presence of the intracardiac mass.

Multimodal imaging plays a critical role not only in establishing the diagnosis but also in enabling ongoing serial monitoring of cardiac masses. cMRI can be used as a powerful tool to differentiate benign from malignant intracardiac masses. Malignant lesions typically demonstrate infiltrative growth patterns, irregular or ill-defined borders, heterogeneous signal intensity, and evidence of central necrosis. They often show early enhancement and heterogeneous late gadolinium enhancement due to neovascularization and necrosis. In contrast, benign masses are usually well-circumscribed, homogeneous, and lack invasive features. In this case, the infiltrative nature of the mass with central necrosis and heterogeneous enhancement strongly favored a malignant etiology despite nondiagnostic biopsy findings. Although the biopsy was inconclusive, which was not surprising given the location of the mass, a surgical biopsy was unlikely to alter management due to the high clinical suspicion for a metastatic cardiac mass from her colorectal adenocarcinoma. Additionally, the initial presence of a biopsy-confirmed anterior mediastinal metastatic adenocarcinoma is consistent with systemic neoplastic disease. PET-CT imaging further supported this diagnosis by revealing a hypermetabolic intracardiac lesion. Lastly, the reduction in mass size following systemic chemotherapy in the past strongly reinforces the likelihood of a metastatic cardiac tumor. Alternative diagnoses were considered. Infective endocarditis was deemed unlikely due to the absence of infectious symptoms or evidence of bacteremia. Hypertrophic or infiltrative cardiomyopathy is less likely given her imaging findings.

Management of cardiac metastases is challenging and typically involves multidisciplinary care. Due to the condition's rarity, there are no standardized guidelines for treatment [4]. Certain regimens, including FOLFIRINOX [4], capecitabine with bevacizumab [7], and FOLFOX with panitumumab [8], have demonstrated effectiveness in isolated case reports. Our patient responded to systemic chemotherapy with significant tumor regression. Surgical intervention may be a viable option in cases of extensive cardiac involvement when performed by experienced surgeons at high-volume centers. It should be considered when it has the potential to improve quality of life, alleviate symptoms, or positively impact prognosis. In this case, surgery was deferred due to the presence of extracardiac disease and the absence of significant cardiac symptoms that would be improved by resection.

## 4. Conclusions

Cardiac metastases are more common than primary cardiac tumors. Clinical presentations may vary depending on the tumor's size and location. Differentiating metastatic disease from competing diagnoses, including thrombus or endocarditis, is important in defining an appropriate therapeutic plan. Multimodal imaging is important in characterizing malignant disease in the heart. Treatment will often require a multidisciplinary approach. Even though complex cardiac surgical intervention was feasible at a high-volume center, it was deferred due to metastatic disease. This case serves to demonstrate success associated with medicinal approaches in metastatic disease with extensive cardiac involvement.

## Data Availability

The data that support the findings of this study are available from the corresponding author upon reasonable request.
